# Identification of interacting neural populations: methods and statistical considerations 

**DOI:** 10.1152/jn.00131.2023

**Published:** 2023-07-19

**Authors:** Robert E. Kass, Heejong Bong, Motolani Olarinre, Qi Xin, Konrad N. Urban

**Affiliations:** ^1^Machine Learning Department, Carnegie Mellon University, Pittsburgh, Pennsylvania, United States; ^2^Neuroscience Institute, Carnegie Mellon University, Pittsburgh, Pennsylvania, United States; ^3^Department of Statistics & Data Science, Carnegie Mellon University, Pittsburgh, Pennsylvania, United States

**Keywords:** cross-population coupling, information flow, latent drivers, statistical issues

## Abstract

As improved recording technologies have created new opportunities for neurophysiological investigation, emphasis has shifted from individual neurons to multiple populations that form circuits, and it has become important to provide evidence of cross-population coordinated activity. We review various methods for doing so, placing them in six major categories while avoiding technical descriptions and instead focusing on high-level motivations and concerns. Our aim is to indicate what the methods can achieve and the circumstances under which they are likely to succeed. Toward this end, we include a discussion of four cross-cutting issues: the definition of neural populations, trial-to-trial variability and Poisson-like noise, time-varying dynamics, and causality.

Listen to this article’s corresponding podcast at https://jneurophysiol.podbean.com/e/jnp-micro-podcasts-four-questions-robert-kass/.

## Graphical abstract

**Figure:**
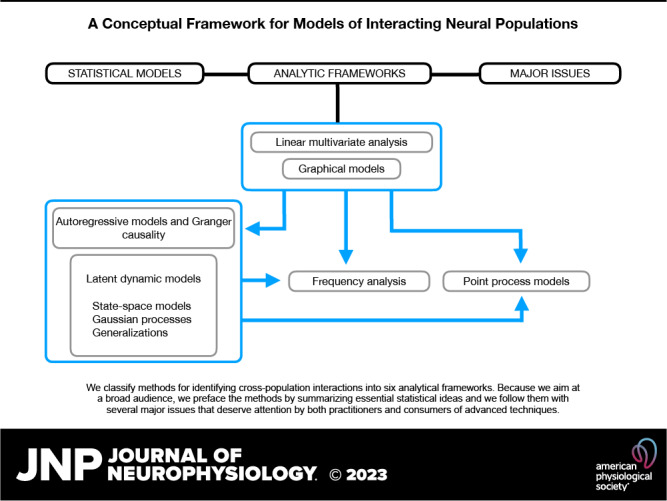

## INTRODUCTION

Neural circuits are described in terms of anatomical areas and subareas, including, when relevant, consideration of cortical structure within and across areas (1–6). Although early investigations tended to focus on the function of neurons within a particular area or subarea, multielectrode data recordings have opened up new possibilities for scientific investigation (7–11), and emphasis has shifted from individual neurons to populations (12–15). Thus, brain physiology is widely conceptualized in terms of interacting neural populations, and it has become important to be able to provide statistical evidence of cross-population neural activity. Here, we review methods for identifying interactions across two or more populations, which are typically populations residing in distinct areas or subareas. We concentrate on spike trains and local field potentials (LFPs, which represent local bulk population activity, Refs. 16, 17, and 18 p. 588), but many of the techniques are used (or could be used) with calcium imaging, EEG/MEG, or fMRI. Throughout, we discuss stereotypical, repeated-trial experiments, though the methods may also apply to peri-event time intervals in experiments involving freely behaving animals (19–21).

Several useful overviews have appeared already, each with its own focus (22–25). Such articles serve differently diverse audiences in neurophysiology, as authors strike some balance of emphasis between conceptual underpinnings and technical description. We have chosen to organize this article around a small number of analytical frameworks that are useful in examining the rich data sources now available, but while we classify methods for identifying cross-population interactions, our main purpose is to highlight the relevance of several fundamental statistical issues. Every method we mention assumes that neural populations are part of a circuit that accomplishes something; yet each time a circuit does so, measured values are different. These two aspects of experimental reality are often described in terms of “signal” and “noise,” with circuit accomplishment being portrayed evocatively using concepts such as “coding” and “information” (historical remarks may be found in Section 1 of Ref. 26). As a consequence of physiological variation, conclusions are based on some definition of consistency in the patterns of neural activity across repetitions, which requires both a notion that the patterns are relevant (e.g., to the flow of information) and a demonstration that the consistency passes a statistical standard for evidence. Our discussion aims to guide thinking about the linked criteria of physiological relevance and statistical standards.

The many ways that noisy data can provide evidence about the function of neural circuits are likely to be of interest to readers with a wide range of backgrounds; this includes a range of comfort with mathematically technical material. Although targeting an audience having a specific assumed level of familiarity with mathematical and statistical concepts would make exposition easier, we believe our overview can be of use to diverse readers. By emphasizing high-level motivations and concerns, we hope to indicate what the methods can achieve and the circumstances under which they are likely to succeed so that experimental investigators can identify scientific questions where these tools can be used to advantage. To maintain wide accessibility, we have chosen not to use mathematical equations. Without equations and more extensive explanations of procedures, those having technical backgrounds may find our very brief summaries inadequate, but rigorous definitions can be retrieved quickly from cited references and other sources.

As an example of our presentation mode, we cite some of our own recent research, which began with the assumption, made by numerous studies, that behaviorally relevant information is transmitted across parts of the brain through transient bursts of activity in neural populations. If population bursts are indeed important, their timing should reveal coordinated activity: on a trial-by-trial basis, the time of a burst in one population should be related to the time of a corresponding burst in a downstream population. A study by Chen et al. (27) computed, across multiple simultaneously recorded populations, on a trial-by-trial basis, the times at which the population’s maximal firing rates occurred. Some of the most remarkable results are given in Fig. 11. Although we will say more about this work in *Point Process Models*, we will not describe it in detail. Our aim, instead, is to direct readers to the underlying presumption that population bursts represent the propagation of information, the methods’ suitability for describing timing with millisecond precision, its use of a broadly applicable statistical framework involving latent variables, and the key components of the approach that apparently drive its ability to find strong representations of interaction.

**Figure 1.**
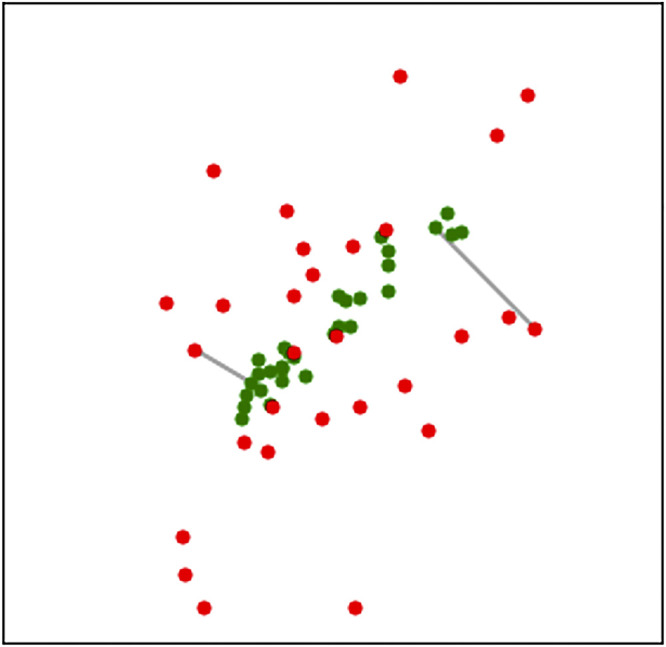
Attenuation of correlation. Green dots show data sampled from a normal distribution for which there is a relatively high correlation between two variables (confer Fig. 11*C*; units along the axes are not shown here because they are arbitrary). Red dots are generated by adding noise to each of the variables represented by green dots (conceptualized as similar to Fig. 11*B* but less extreme). Gray lines show the mapping from the original green dot to the corresponding red dot, once noise has been added. The correlation between green dots is much higher than the correlation between red dots.

We emphasize these sorts of considerations by sandwiching our list of methodological approaches, in analytic frameworks, between general statistical discussions in statistical models and important issues. In statistical models, we outline a few essential statistical concepts, including latent variables, which appear in many models of cross-population coupling to represent idealized, shared “drivers” of activity (illustrated in Fig. 5). In analytic frameworks, we categorize data analytic methods used to describe interacting populations according to six major frameworks (see Fig. 4). In summarizing them, we aim to capture the main ideas behind their use so that their purpose in this context can be grasped, in rough terms, by nonexperts. The first four general frameworks are linear multivariate analysis, graphical models, autoregressive models (especially for Granger causality), and latent dynamic models. Our last two general categories of methods are more specialized approaches based on frequency analysis and point processes, the former being applied mainly to LFPs (and similar continuously varying quantities measured repeatedly across time) and the latter being suitable for modeling spike trains. In important issues, we highlight a series of features of neurophysiological data that occur frequently and that every investigator should consider, starting with the definition of a neural population, which involves neural diversity, combined with haphazard neural sampling, and the redundancy of neural signals. There are also statistical difficulties associated with large numbers of signals. We then discuss trial-to-trial variability, where Poisson-like variation can easily obscure interesting relationships, while statistical techniques can uncover them (as illustrated in Fig. 1, Fig. 11, *B* and *C*, and Fig. 1). A ubiquitous concern is time-varying dynamics, which can invalidate many common methods in principle, suggesting care in practice. We end our call to attention with remarks on causality and share a few closing thoughts in our discussion.

## STATISTICAL MODELS

### Models and Parameters

It is not too hard to summarize several important teachings from the field of statistics, as in the *Ten Simple Rules* in the article by Kass et al. (28) or the 14-page explanation of the “statistical paradigm” in the book by Kass et al. (29, Chapter 1). Arguably the most fundamental quality of data analytic methods informed by the field of statistics is that they are defined and studied by introducing statistical models. That is, rather than summarizing data based solely on intuition, or heuristics, according to the statistical paradigm one begins by defining a model that uses probability distributions to describe the regularity and variability in the data. This may be written in schematic short-hand, as

(*1*) $$\text{observation}\overset{\text{combines}}{\equiv }\text{signal}\overset{\text{with}}{+}\text{noise},$$

where the way signal and noise are combined, through the statistical model, may be either simple or complicated. Importantly, when a statistical method is derived by assuming a particular model, it can be evaluated using a variety of other, different models. This is a reliable way to check the robustness of a method against departures from assumptions. In the machine learning literature, statistical models are often called “generative models” (indicating that they can generate artificial data presumed to be similar to real data).

The probability distributions used in statistical models are specified by probability density functions, which depend on theoretical quantities, known as parameters. For example, a normal (Gaussian) distribution has a mean and standard deviation as parameters. When there are only a few parameters, a model may be relatively easy to understand but it restricts possibilities for data variation. On the other hand, when a very large number of parameters is used (in theory, even infinitely many) models can be very flexible.

It is important to distinguish between data-based quantities and their model-based theoretical counterparts. For example, a sample mean is computed from data, and its model-based counterpart could be the parameter representing the mean of a normal distribution; the latter is often called a population mean, but here we will use the term *theoretical* instead of “population” for reasons articulated in the book by Kass et al. (29, section 3.2.2), and at greater length in the article by Kass (30). Statistically, the sample mean would be considered an estimate of the theoretical mean. Many neurophysiologists become comfortable with data-based quantities, such as a firing rate or a spike count correlation, without considering a statistical model. This is fine until the variation in the data summary is sufficiently large that it creates nontrivial uncertainty—but immediately a question arises: uncertainty about what? The answer that has remained useful since its introduction by R.A. Fisher 100 years ago (31) invokes a theoretical counterpart to the data summary, and it is defined in terms of a statistical model. Evaluation of the strength of evidence, usually based on confidence intervals or significance tests (or Bayes factors, see Ref. 32), relies on this fundamental conception. Concerns about the validity of some statistical manipulation of the data can only be addressed by an examination of the procedure under conditions specified by a model together with a data-informed judgment of the suitability of that model.

### Dimensionality and Multivariate Models

Because parameters provide numerical labels to identify specific members of a family of probability distributions that make up a model, there is (for most standard models) a well-defined notion of the dimension of the model: it is the number of freely ranging univariate parameters used to identify a member of the family (see Theorem 2.3.1 and Section 7.3 of Ref. 33; subtleties can arise, see Ref. 34). Similarly, sets of random variables that represent data have an associated dimension. Activity across *n* recordings would, unless somehow constrained, typically be assumed to vary freely in an *n*-dimensional space, that is, a set of values represented by a vector with *n* coordinates. When a variable *X*_1_ can be written in terms of two other variables *X*_2_ and *X*_3_, the collection (*X*_1_, *X*_2_, *X*_3_) is not freely ranging in a three-dimensional space; instead, it has two dimensions (at most; if the three variables are multiples of each other then there is only 1 dimension of variation). This is the source of the terminology “degrees of freedom” in statistics. That kind of mathematical redundancy is not difficult to accommodate.

The situation becomes more subtle when statistical redundancy is considered. Statistical redundancy would occur if a variable *X*_1_ (a random variable, following a probability distribution) was highly correlated with some combination of variables *X*_2_ and *X*_3_. In this case, it is common to introduce an empirical rule that uses some measure of correlation together with a threshold which, when exceeded, lumps the high correlation case together with exact mathematical dependence by deeming the collection (*X*_1_, *X*_2_, *X*_3_) again to be lower dimensional. Similar reasoning applies when there are *n* variables having variation concentrated predominantly in a space of lower dimension *k*. Statistical redundancy arises in neural activity when the *n* recordings cannot vary separately in response to a stimulus or in contributing to behavior. This is analogous to finger kinematic redundancies during reaching tasks, where hand physiology constrains the fingers so that they cannot move independently (e.g., Refs. 35 and 36). As the number of recordings increases, some degree of statistical redundancy becomes likely. In the case of individual neurons, this is likely due to the redundancy of inputs across a population combined with a limited sampling of stimuli or behaviors in the experiment. For LFPs, there is also a well-documented spatial correlation in neighboring recordings (17, 37). Consideration of dimensionality has been used to interpret population responses (38–41) and cross-population communication (see Refs. 42 and 43 and references therein).

The formal framework for analyzing the covariation among *n* variables begins with their variance matrix, which is defined by the *n* standard deviations together with the correlations of all pairs of variables. The most common method of dimensionality reduction involves a revealing reexpression of that matrix: the coordinates representing the *n* variables are rotated to define new, uncorrelated variables; the coordinate direction corresponding to the new variable that has the largest standard deviation represents the direction of maximal variance among linear combinations of the original variables; the representation of data along this coordinate is called the first principal component; and the complete decomposition using multiple components becomes Principal Component Analysis (PCA). Factor analysis relies on similar reexpressions (it is illustrated conceptually in Fig. 5). It is easy to find good explanations of these methods, and basic intuitions appear in the article by Cunningham and Yu (39). As we discuss in *High dimensionality*, redundancy tends to become more pronounced when a variance matrix is estimated from data and *n* is large.

**Figure 2.**
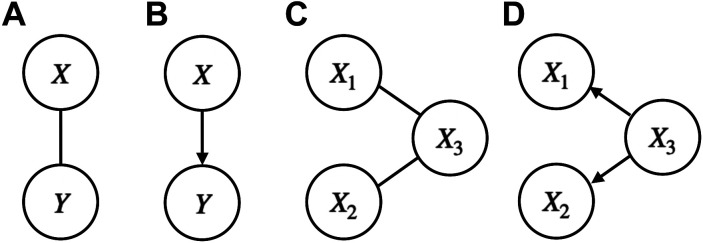
Correlative and predictive graphs. *A*: the random variables *X* and *Y* are correlative, in the sense of the ordinary (Pearson) correlation or some other measure of association. *B*: the variable *X* can be used to predict *Y*. *C:* in general, when a pair of nodes representing two random variables are connected by an edge (a line), the two random variables remain dependent after conditioning on all other random variables. Here, *X*_1_ and *X*_2_ are conditionally independent given *X*_3_. If these variables represented activity in different brain areas, and if we were to assume that no other brain areas are relevant, then, based on these activity variables, any apparent interaction between areas 1 and 2 would be due to the separate interactions of area 1 with area 3 and area 2 with area 3. *D*: variable *X*_3_ predicts each of *X*_1_ and *X*_2_, and these predictive relationships could result in an observed “spurious” association between *X*_1_ and *X*_2_. Although pictorial diagrams such as in *B* and *D* are commonly used in neurophysiology (where the variables would be replaced by brain locations) to suggest neural transmission or information flow, they are infrequently based on causal evidence (in the sense specified by statistical causal inference) and are more commonly predictive. In general, each variable depicted in the figure could itself be a random vector rather than a scalar random variable.

To highlight and clarify the distinction between pairwise and multivariate dependence, let us step through the case of three variables *X*_1_, *X*_2_, *X*_3_, where it is possible to have the correlations of *X*_3_ with each of *X*_1_ and *X*_2_ “explain” a correlation between *X*_1_ and *X*_2_ in the sense that if *X*_3_ were held constant the correlation between *X*_1_ and *X*_2_ would disappear, that is, the partial correlation of *X*_1_ and *X*_2_ after conditioning on the value of *X*_3_ would be zero. Figure 2*C* illustrates this situation, while Fig. 2*D* adds arrows to show what is usually called a spurious correlation between *X*_1_ and *X*_2_, due to the *confounding* variable *X*_3_. In the case of Gaussian (normal) random variables, just as *X*_1_ and *X*_2_ are independent when their correlation is zero, *X*_1_ and *X*_2_ become *conditionally independent* after conditioning on *X*_3_ when their partial correlation given *X*_3_ is zero (Fig. 2*C*). When the variables *X*_1_, *X*_2_, *X*_3_ represent summaries of neural activity in three areas, if we were to consider all other areas to be irrelevant, this would indicate that an observed interaction of *X*_1_ and *X*_2_ (in the sense of correlated activity) would be due entirely to the separate interactions of *X*_1_ with *X*_3_ and *X*_2_ with *X*_3_. Put differently, there is a dependence that is unique to the pair *X*_1_ and *X*_3_ and also to the pair *X*_2_ and *X*_3_, but there is no dependence that is unique to the pair *X*_1_ and *X*_2_.

Graphical diagrams such as those in Fig. 2 are commonly used in neuroscience, with brain areas replacing the variable names inside the circles, but the interpretation of the connecting lines or arrows between the circles depends on context. In graphical models the circles are called “nodes” and the connecting lines are “edges.” *Probabilistic graphical models* represent probabilistic dependencies, with Fig. 2*C* being the simplest nontrivial probabilistic graphical model. For a probabilistic graphical model, the absence of an edge between two nodes indicates the independence of the two corresponding variables after conditioning on all the rest of the variables (Ref. 44, pp. 429–440 and 939–940). As reviewed in *Graphical models*, graphical models can represent interesting dependence relationships across multiple populations. They are also a starting point for network analysis.

The graphical models in Fig. 2, *A* and *C* are *undirected*. When the edges are replaced by arrows, as in Fig. 2, *B* and *D*, the graph becomes *directed*. Directed graphs are highly suggestive, but converting an undirected relationship based on correlation structure to a directed relationship requires additional information. In fact, it is not hard to create an example in which Fig. 2*C* represents the correlation structure but, rather than the correlation of *X*_1_ and *X*_2_ being due to *X*_3_ the relationship is reversed: the combination of *X*_1_ and *X*_2_ produces *X*_3_, resulting in a graph as in Fig. 2*D* with the arrows pointing toward, rather than away from *X*_3_. In neuroscientific applications, area-to-area arrows are generally based on some assessment of association (correlation and its generalizations) supplemented by consideration of timing. In most cases, however, arrows representing timing should be interpreted as predictive rather than causal, a point mentioned in *Autoregressive models and Granger causality* and discussed in *Causality*.

Although only under rare circumstances can causal claims be justified based on partial correlation (and analogous assessments of association) in conjunction with timing information, they remain useful more broadly: when the partial correlation of *X*_1_ and *X*_2_ given *X*_3_ is much smaller than the original correlation of *X*_1_ and *X*_2_ (before conditioning on *X*_3_), the interpretation is that the separate interactions reflected in the statistical associations of *X*_3_ with *X*_1_ and *X*_3_ with *X*_2_ contribute substantially to the interaction reflected in the observed association of *X*_1_ with *X*_2_.

**Figure 3.**
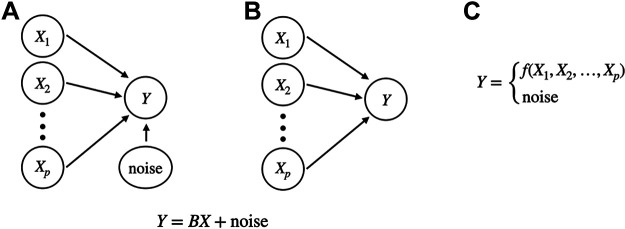
Regression models. *A* and *B*: two pictorial representations of the linear regression model, having the equation entered below the two diagrams. In *A*, the noise variable is entered explicitly, but diagrams like that in *B* are often used, with the noise entering implicitly. Both diagrams show the dependence of *Y* on the set of variables *X*_1_, *X*_2_,…, *X_p_*. Note that *B* would be considered the same as Fig. 2*B*, if *X* in that figure were the vector *X =* (*X*_1_, *X*_2_,…, *X_p_*) used here. *C*: diagram of generalized regression emphasizing that a function of the *X* variables is combined, somehow, with noise to produce *Y*. In the linear regression model noise is added to the linear relationship that determines the regularity in *Y*, i.e., the “signal.” In *C*, the combination can be nonlinear and nonadditive, but a probability distribution represents variation that would be considered noise. The diagram in *B* may also be used for the generalized regression case when the arrows indicate dependence but need not represent linear dependence.

The study by Chen et al. (27) analyzed data from visual area AL, in addition to V1 and LM as shown in Fig. 11. The three variables denoted here as *X*_1_, *X*_2_, *X*_3_ became summaries of activity in three populations for Chen et al. (27). This is one common strategy, and it often employs latent variables, discussed in *Latent variables*. An alternative is to consider the predictability of each recording (spike train or LFP) from the many recordings in the other populations. This becomes a regression approach, illustrated in Fig. 3, *A* and *B*, but it is usually carried out using some form of generalized regression (emphasized in Fig. 3C). A simpler possibility, for two populations, is to examine pairs of individual recordings, one in each population, for many pairs (as in Fig. 2*B*). The methods we review include examples of all three approaches.

Concerning terminology, it can be cumbersome to distinguish between scalars and vectors. Thus, in some cases, when we speak about a variable, as in referencing a node in a graphical model such as those shown in Fig. 2, the “variable” will actually be a vector. We trust that this will not cause confusion.

### Stochastic Processes

In probability theory, a stochastic process (in time) is a collection of variables *X_t_*, with one variable for each value of time *t*. When *t* takes values 1,2,3,… the process is called a time series (see *Autoregressive Models and Granger Causality*). Thus, LFPs are analyzed as time series. For point processes (*Point Process Models*), in theory, time is continuous, but in practice, spike trains are observed at discrete time values, with resolution such as 1 ms, so that the spike train data form binary time series (spike or no spike in each time bin). Similarly, in theory, Gaussian processes (GPs) (see *Gaussian processes*) are continuous, but they are applied to analyze time series of spike counts. A technical point is that a probability distribution for a stochastic process must specify all possible multivariate joint distributions, which leads to simplifying assumptions.

### Latent Variables

Especially important in population analysis is the idea that activity within a population may have a commonality conceptualized as a “hidden driver,” often considered to represent a “hidden state.” These are known as latent variables. The adjectives “latent” and “hidden” are used because latent variables do not correspond to any measured quantities; rather, they are theoretical abstractions introduced to construct a concise and intuitive model of covariation (see Ref. 45, Ref. 29, Section 16.2, and Ref. 44, pp. 337–420). Latent variables may appear in models as parameters, or their effects may depend on parameters through some mathematical relationship. We illustrate some common latent variable structures in Fig. 5 and discuss them in the context of specific models below.

#### Theoretical firing rates as latent variables.

In spike train analysis, spike counts can be converted into observed firing rates. Within a statistical model for neuron spiking, corresponding to an observed firing rate there is a theoretical firing rate, determined by one or more parameters; typically, this theoretical firing rate, and its dependence on experimental variables characterizing a stimulus or behavior, would be the focus of attention. When the fluctuation in a theoretical firing rate is considered to be stochastic, depending on some set of random variables (representing the variation in stimuli or behaviors, etc.), it becomes a latent variable. A simple example is the use of “high” and “low” firing rates in a single-neuron model to capture bursting (Ref. 46, which is example 16.3 in Ref. 29). In the conceptually simplest version of that example, the model would have the structure of Fig. 5C, where *Z^t^* becomes the high or low firing rate at time *t* and *Y^t^* becomes the corresponding spiking behavior at time *t* (spike or no spike). Studies of neural variability sometimes examine theoretical firing rates instead of spike counts because the latter includes Poisson-like noise. The Poisson-like noise can be effectively reduced through the use of a statistical model that separates the theoretical firing rate from the Poisson-like noise and reports estimates of theoretical quantities, such as firing rate correlation instead of spike count correlation (47, 48). This use of firing rates typically results in much higher values of correlation between pairs of neurons, which in statistics is called “correction for attenuation” (49). We return to Poisson-like noise in *Point Process Models* and *Trial-to-Trial Variability and Poisson-like Noise*, but the idea behind the attenuation of correlation is illustrated in Fig. 1 whereas Fig. 11*C*, in comparison with Fig. 11*B*, illustrates the benefit of removing Poisson-like noise for identifying cross-area interactions.

#### Latent drivers and hierarchical interactions.

A prominent use of latent variable models is to provide a parsimonious representation of the redundancy in neural signals mentioned in *Models and parameters* by restricting the variation of interest to a subspace of lower dimension than that of the observations themselves. Such dimensionality reduction is the purpose of factor analysis (see Fig. 5), where every measured variable is assumed to arise as a linear combination of some comparatively small number of latent variables (assumed independent and standard normally distributed), plus noise. In addition, PCA, and the closely-related canonical correlation analysis (CCA, see *Linear Multivariate Analysis*), can be derived as estimation procedures for latent variable models known as probabilistic PCA and CCA (pPCA, pCCA; see Ref. 44, Sections 20.2.2 and 20.2.8.3). The resulting latent variables become conceptualized as latent drivers of neural activity, pictured with arrows in Fig. 5. As we review in *State-space models* and *Gaussian* *processes*, cross-area analysis may be simplified by assuming one or more drivers of activity in each area: although the number of distinct spike train or LFP recordings may be large, the number of separate populations (typically from anatomically distinct areas) is much smaller, which suggests identification of cross-population interactions per se ought to be more manageable.

To describe situations in which interactions among neurons, or field potentials, might occur either within a particular population or across two or more populations, it may be helpful to use the term *hierarchical interactions*. Here, we refer to statistical behavior rather than the widely discussed anatomical and physiological hierarchical structure of the cortex (4, 50–52). In this conception, cross-population interactions are summarized with low-dimensional latent variables (latent vectors), while within-population activity is determined partly by one or more relevant latent variables and partly by other factors. Two approaches that have been used to capture hierarchical interactions are Gaussian Process Factor Analysis (GPFA), which is discussed in *Gaussian processes*, and analysis using Bayesian hierarchical models (see Refs. 53–55; additional references in Ref. 56; and Ref. 29, Chapter 16). An example of the latter is the model in the study by Chen et al. (27), which produced Fig. 11. The general idea, however, is not tied to a particular methodology.

Regardless of the modeling details, it is worth noting that when cross-population interactions are the focus of interest, parameters representing within-population interactions may be treated differently than parameters that identify cross-population interactions. In the statistical literature, necessary but uninteresting parameters are often called “nuisance parameters” (see Ref. 57, Chapter 4 and Ref. 58, Chapter 25). In the case of a normal distribution, interest typically focuses on the mean, with the standard deviation becoming a nuisance parameter. This piece of terminology is helpful conceptually and, in practice, statistical methods focused on parameters of interest are, in some circumstances, comparatively insensitive to errors in identifying values of nuisance parameters. In cross-population studies, parameters controlling variation *within* populations (as opposed to *across* populations) would typically be considered nuisance parameters. For example, in their assessment of beta-frequency amplitude coupling between the prefrontal cortex (PFC) and visual area V4 during a visual memory task, the study by Bong et al. (59) (described briefly in *Gaussian processes*) treated the correlation structure among LFPs within each area as a high-dimensional nuisance parameter: even though the estimate of a general correlation structure among nearly 100 LFPs cannot be expected to be highly accurate, probable inaccuracies were considered unlikely to undermine the evaluation of cross-population coupling.

## ANALYTIC FRAMEWORKS

The data analytic techniques we describe may be considered, interpretively, to be ways of arriving at pictorial representations like those in Fig. 2. One broad distinction is between directed and undirected graphs (Fig. 2, *B* and *D* vs. Fig. 2, *A* and *C*). The simplest case, in Fig. 2, *A* and *B*, distinguishes regression from correlation, with *X* being used to predict *Y* in Fig. 2*B*. In fact, in this context, *X* variables are often called “predictors.” This choice of words suggests that *X* precedes *Y*, and regression across time, carefully defined, can give directional arrows in the sense of prediction (though the “predictor” terminology is used even in cases that do not involve time to indicate that the *X* variables *could* be used predictively). Figure 3 provides a more detailed set of diagrams for regression.

In thinking about the many analytical approaches that can provide evidence for either correlative or predictive statistical dependence, it is important to remember that there are many ways multiple variables might interact, and as the number of variables grows it quickly becomes impossible to learn solely from data an accurate probability model that describes all the potentially complicated interactions. To make progress, some simplifying assumption is necessary. In the most common statistical contexts, it is assumed that all the variables are independent of each other: in this case, the probability density function for all of them together becomes the product of the individual-variable probability density functions. That, however, does not allow for statistical dependence (as measured by correlation, for example). An intermediate simplification is to assume some form of conditional independence, as illustrated in Fig. 2*C*. When graphs are used to supply dependence structure (as in Fig. 2*C*), they define probabilistic graphical models. The resulting probability density function, for all the variables together, can then be factored into simpler components based on conditional independence, which makes computation and statistical inference much more manageable.

The methods we summarize make use of these considerations: they are based on statistical models that define correlative or predictive relationships by imposing structure that often makes use of conditional independence (assuming it either explicitly or implicitly, or by learning it from data). Our decomposition into six categories reflects in part the structure of the data and in part the relationships a method seeks to uncover. Autoregressive models, latent dynamic models, and frequency analysis (our third, fourth, and fifth categories) are all relevant to time series, i.e., substantial numbers of repeated measurements made at different times, sequentially, such as every millisecond (LFPs are time series). Point processes (the sixth category) are specific to spike trains and are especially valuable when precise timing of changes in firing rate could be important, so that counts in wide time bins might miss interesting effects. Time series can be analyzed by examining either behavior across time or oscillatory behavior for particular frequencies. Time-domain and frequency-domain methods are interrelated, sometimes they are used together, and sometimes time-domain methods consider particular frequencies whereas frequency-domain methods can involve dynamic evolution across time. Nonetheless, autoregressive models, latent dynamic models, and point process models emphasize time-domain methods.

**Figure 4.**
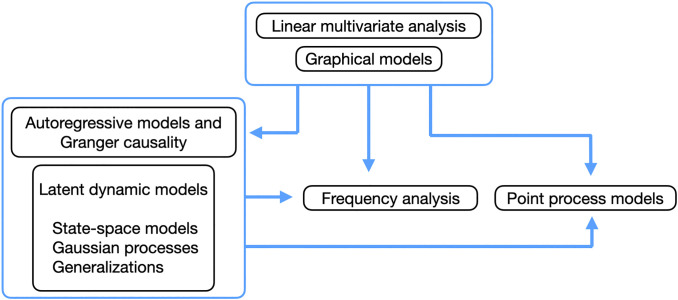
Our six categories of methods (in black boxes) are arranged to display some of their relationships. The blue box surrounding linear multivariate analysis and graphical models indicates that they could be considered part of one subject devoted to relationships among multiple variables. Similarly, autoregressive models are important components of many latent dynamic models and, together, the models within their blue box describe approaches to analyzing time series. Arrows indicate the use of one kind of model within the context of another. For example, many types of latent dynamic models have been used with point processes to analyze multiple spike trains.

Figure 4 illustrates some of the relationships among the six categories. The first collection of methods we describe falls under the heading of linear multivariate analysis, which (usually without the modifier “linear” we have inserted for emphasis) is a branch of statistics first developed before high-speed computers, when it was essential to have mathematically tractable solutions to problems, and the models were based mainly on multivariate normal distributions. In addition to remaining relevant, it serves to introduce some more general ideas. Graphical models (the second category) could be considered part of multivariate analysis, which is why Fig. 4 puts those first two categories of methods inside their own box. Importantly, graphical models can be applied to many kinds of data, especially using special classes of probability distributions called exponential families, which in some important ways can be considered analogous to multivariate normal distributions.

Within each category, we will list several main approaches and tools, but we can describe them here only very briefly. Some of the different methods may have different purposes as, we hope, will be clear. It is very common, however, to find several different statistical methods that claim to solve the same underlying problem because they impose different structural assumptions, as we will indicate; the choice among the alternatives becomes a judgment of which structures seem most relevant, or most intuitive, or most convenient in a given context. Our primary goal is to provide an organizational and conceptual view of the data-analytic questions being answered by available methodologies.

### Linear Multivariate Analysis

When activity is recorded by multiple electrodes (e.g., yielding multiple spike counts) in two areas, an initial question is whether an increase in activity in one area tends to correspond to an increase in activity in the other. The simplest assessment of covariation across recordings, such as spike counts, from only two electrodes, one in each area, is the ordinary (Pearson) correlation. The existence of such a relationship is depicted in Fig. 2*A*. To summarize the covariation across many recordings, a standard approach represents the variables in one area by a vector *X* and those in the other by a vector *Y* and then finds the linear combinations of components of these vectors that have the greatest correlation, known as the canonical correlation; the vectors formed by the linear combination coefficients are the canonical vectors. When the canonical correlation is sufficiently large (and statistically significant) this can give a definition for the connecting line in Fig. 2*A*, where *X* and *Y* are vectors rather than scalars. The procedure is elaborated by removing from each of *X* and *Y* the variation due to their respective canonical vectors (by orthogonalization) and then treating the pair of residual variation vectors as before yielding a second correlation, which becomes the second canonical correlation, and a second canonical vector that is orthogonal to the first. The process may be repeated some number of times to produce Canonical Correlation Analysis (CCA). Semedo et al. (60) used CCA to analyze interactions between both V1 and V2 and V1 and V4, based on spike counts, applying time delays between areas to draw conclusions about feedforward and feedback interaction. When applied at multiple time points, the linear combinations in CCA, determining the relative weights given to particular neurons, typically vary from time point to time point, which seems undesirable. Rodu et al. (61) developed a windowing method to stabilize the solutions; they applied it to describe time-varying interactions, including time lags, between PFC and hippocampus during a memory task based on multiple LFPs recorded from each area. Multiset CCA (62) extends CCA to multiple groups (corresponding to multiple areas).

An alternative to CCA, known as reduced-rank regression (RRR), treats the two areas asymmetrically, labeling the activity in the two areas by *Y* and *X* and regressing *Y* on a dimension-reduced version of *X*. Ordinary least-squares regression finds the closest representation of *Y* within the space defined by linear combinations of the variables making up *X* (closest in the Euclidean sense of least squares). RRR takes the additional step of requiring the solution (defined by the *X* variables) to be of a lower dimension. Semedo et al. (63) used RRR to predict the activity of V2 neurons from a low-dimensional representation of activity among V1 neurons, which they called a “communication subspace” (confer Ref. 25). Partial least squares (PLSs; see Ref. 44, Section 20.2.8.2) is similar to RRR, but in determining a low-dimensional version of *X* to predict *Y*, it takes into account the variability of *X*. Ames and Churchland (64) suggested that PLS is a suitable tool for analyzing the degree to which contralateral and ipsilateral brain activity predict arm motions.

In applying these linear multivariate techniques, a concern, ubiquitous in statistics, is the inherent arbitrariness in the choice of a criterion that produces a method. In the case of canonical correlation, the maximization of correlation is clearly simple and intuitive, but should not be considered compelling. On the other hand, for the communal enterprise of science, there are big advantages to using well-established methods. Similarly, although linearity is, in principle, restrictive, the noisiness of the data together with modest sample sizes often precludes the identification of nonlinear relationships, and even when some nonlinearity is detected, its effect on results may not change conclusions.

**Figure 5.**
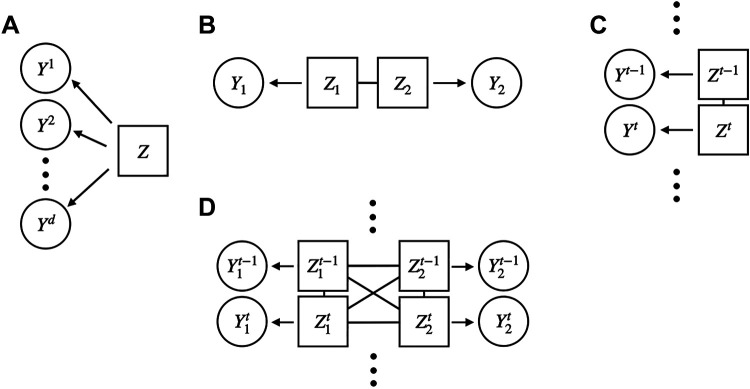
Latent factor models. *A*: the latent variable *Z* affects each of the scalar components of *Y* = (*Y*^1^, *Y*^2^,…, *Y^d^*). In a one-dimensional factor analysis model each component of *Y* is a scalar multiple (often called a weight) of the scalar random variable *Z* (which is normally distributed), and noise is added, independently, to each component. More generally, factor analysis takes *Z* to be a random vector. *B*: in pCCA (in the version used by Ref. 65) a pair of correlated factor variables *Z*_1_ and *Z*_2_ affect the respective vectors *Y*_1_ and *Y*_2_ as in factor analysis; the estimated correlation of *Z*_1_ and *Z*_2_ is equal to the canonical correlation. *C*: in GPFA a latent time series of multivariate Gaussian processes *Z*^1^, *Z*^2^,…, *Z^t^*^−1^, *Z^t^*,… affects the vector time series *Y*^1^, *Y*^2^,…, *Y^t^*^−1^, *Y^t^*,…, with each *Z^t^* mapping to *Y^t^* as in factor analysis, independently and using the same weights across time. The triple dots above and below the variables are used to indicate the time series nature of the variables. *D*: Ref. 65 extended pCCA (*B*) to time series. The diagram takes a pair of diagrams as in *C* and allows the latent variables to be correlated, as in *B*, both within and across time series at various time lags. (The version of pCCA used by Bong et al., as in *B*, is different than the usual representation that involves a single latent variable *Z*, but it is equivalent in the sense of reproducing CCA.) GPFA, Gaussian Process Factor Analysis; pCCA, probabilistic canonical correlation analysis.

A more modern development represents classical methods such as PCA and CCA as latent variable models, as mentioned in *Latent Variables* and illustrated in Fig. 5*B*, and then goes on to show that these alternative versions can have practical advantages. Murphy (44, Section 20.2) uses the model-based framework to provide a unified treatment of many methods, including PCA, CCA, factor analysis, PLS, and a variety of nonlinear extensions. As described in *Gaussian processes* (see Fig. 5*D*), Bong et al. (65) used such a model-based framework to summarize interactions across multiple time series of LFPs from PFC and V4.

### Graphical Models

Graphical models represent relationships among variables using nodes connected by edges. When such diagrams represent relationships according to probabilistic graphical models, the absence of an edge between two nodes corresponds to conditional independence (of the variables for those two nodes) given all the variables represented by all the other nodes. The simplest case was discussed with reference to Fig. 2*C* in *Dimensionality and Multivariate Models*; as explained there, for Gaussian graphical models the absence of an edge corresponds to zero partial correlation.

**Figure 6.**
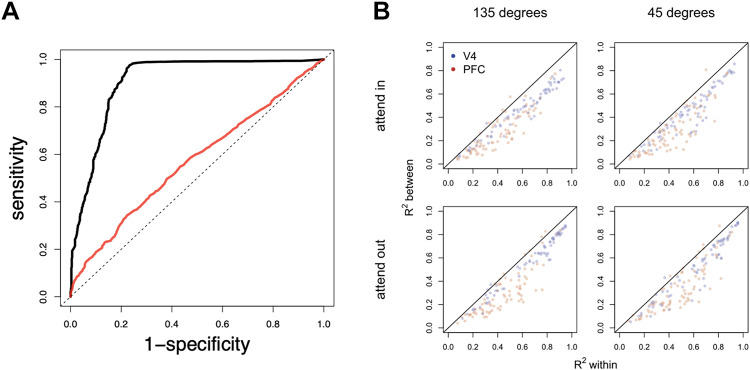
Results for the graphical model of firing rates in the prefrontal cortex (PFC) and V4. *A*: the ROC curve for the usual graphical Lasso (red) is far below the ROC curve for the method of Ref. 66 (black). The ROC curves, which summarize the operating characteristics of the two procedures for identifying zero or nonzero partial correlations, are based on data simulated to be similar to real data (but with known ground truth). The ROC curve plots the probability of (correctly) detecting a nonzero partial correlation, when it is truly nonzero, against the probability of (incorrectly) claiming a nonzero partial correlation when it is truly zero. The curve for an essentially perfect procedure would jump from the lower left corner to the upper left corner (high probability of correct detection, low probability of false claim), to the upper right corner; the curve for choosing by chance alone (equal probabilities of claiming zero or nonzero) would hug the diagonal (dotted line). Thus, the graphical Lasso is only a little better than chance (coin-flipping) in identifying zero vs. nonzero partial correlations while the method of Ref. 66 is much better. *B*: the method of Ref. 66 estimates a variance matrix, from which regression *R*^2^ values are easily calculated. In the four panels the *R*^2^ “between” is plotted against the *R*^2^ “within.” Each dot involves a pair of regressions for a particular neuron’s firing rate (blue if that neuron is in V4, red if it is in PFC); that neuron’s firing rate is regressed either on all the firing rates in the opposite area (between, for the *Y* coordinate *R*^2^ in the plot) or on all the other firing rates in the same area (within, for the *X* coordinate). Four experimental conditions are shown involving two stimulus locations and either attending in or attending out according to whether the locations were in or out of the receptive fields. The plots show a wide range of *R*^2^ values, spanning nearly the whole domain of *R*^2^ across neurons, but those neural firing rates that are more (or less) predictable from the other firing rates within the same area *are also* more (or less) predictable from the firing rates in the other area. The method denoises to correct for attenuation, as in Fig. 1 and Fig. 11*C*. Modified from Ref. 66.

When constructing a graphical model from data, it is important to remember that it is essentially impossible to distinguish a zero partial correlation from one that is very close to zero; the situation is analogous to other probabilistic graphical models. Thus, in employing a graphical model there is an implicit assumption that distinctions between small and zero partial correlations (and related measures of association) are not scientifically meaningful for a specified purpose, a point we repeat in *Causality*. With this reality in mind, the construction of a Gaussian graphical model may be considered a way of estimating a variance matrix. When the matrix is large it is difficult to estimate accurately (because of the cumulative effects from inaccurate estimation of large numbers of correlations), and setting many partial correlations to zero (which amounts to determining a graphical model) effectively removes many of the parameters and can stabilize estimation. Vinci et al. (66) combined a technique for high-dimensional problems, L1 regularization, discussed in *High dimensionality*, with correction for attenuation, discussed in *Theoretical firing rates as latent variables*, to analyze spike counts from V4 and PFC during an attention task. As discussed in *High dimensionality*, Vinci et al. (66), in their study, modified the usual L1 regularization procedure for variance matrix estimation, known as graphical Lasso. Not only did this produce a striking improvement in performance, shown in Fig. 6*A*, but it also produced some interesting results shown in Fig. 6*B*.

**Figure 7.**
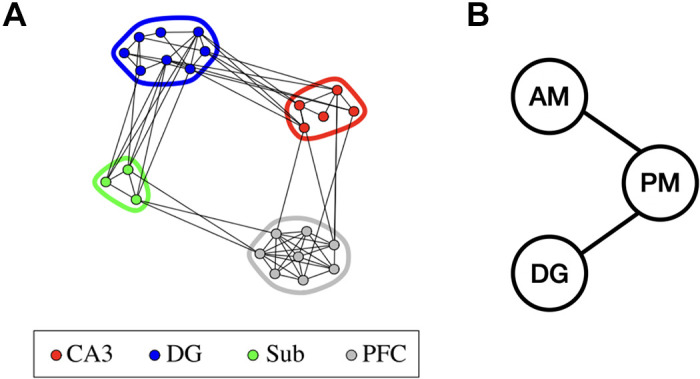
Two kinds of graphical models for area-to-area phase coupling. *A*: the graph showing all significant local field potential (LFP) beta-oscillation phase locking between pairs of electrodes in four areas, after conditioning on phases from all other electrodes. There was no evidence of unique phase locking between the prefrontal cortex (PFC) and the dentate gyrus (DG) that could not be accounted for by phase locking between PFC and either of the two other areas, subiculum (Sub) and CA3, each of which being, in turn, phase locked with DG. Modified from Klein et al. (67). *B*: a portion of the graph found by the method of Ref. 68 applied to theta oscillations across six brain areas; only results for the anteromedial and posteromedial visual areas (AM and PM), and the dentate gyrus (DG) are shown here. Although there was strong evidence of unique coupling (in this case, a latent coherence) between AM and PM and between AM and DG, there was no evidence of unique coupling between AM and DG.

In analyzing recordings across many populations, each node might signify an individual recording, or a population summary of them, or a latent variable representing a population summary. For example, to analyze LFP beta oscillations recorded from 24 electrodes across PFC and the hippocampal areas dentate gyrus (DG), subicullum (Sub), and CA3 during a memory retrieval task, Klein et al. (67) developed a multivariate assessment of phase coupling. In Fig. 7*A* each node represents the phase from a single LFP, for a single electrode, following a global significance test for area-to-area phase locking. Figure 7*B* displays the results of a different analysis of LFP theta oscillations [from the same data set as that analyzed by Chen et al. (27)] based on a latent variable model, where each node represents the phase of a driver of activity within an area.

In *Frequency Analysis*, we will say more about the methods used in Fig. 7. A general point, however, is that the methodology in the study by Klein et al. (67) was based on a class of probabilistic graphical models for which there are direct analogs to Gaussian graphical models: exponential families with two-way interactions (also known as maximum entropy models). The term “interaction” is used in the same way as in the analysis of variance (ANOVA, e.g., Ref. 29, Section 13.2.2). For exponential family models, the absence of a two-way interaction term in the model corresponds to conditional independence; thus, Klein et al. (67) were also able to define an analog of partial correlation for phase coupling. Because each phase angle lies on the unit circle and, mathematically, the product of circles is a torus, the model developed by Klein et al. (67) for the data was an exponential family on a 24-dimensional torus; they called such models “torus graphs.”

### Autoregressive Models and Granger Causality

An important method for identifying interactions across areas from LFPs is Granger causality (69, 70). The idea is simple. A time series of values *X*_1_, *X*_2_, *X*_3_,… at times *t* = 1,2,3,…, is Granger causal of a time series of values *Y*_1_, *Y*_2_, *Y*_3_,… if, for every time value *t*, the past of *X_t_* (values before time *t*) is predictive of *Y_t_*, after taking account of the inherent dependence of *Y_t_* on its own past. One of the first studies that applied Granger causality to LFPs, Brovelli et al. (71), demonstrated, first, that beta oscillations were coordinated across multiple areas during a sensorimotor task (via analysis of coherence, discussed in *Frequency Analysis*) and, second, that the activity in some areas predicted activity in other areas (Granger causality).

The formal definition of Granger causality involves autoregressive processes, which are important in their own right. A time series taking values *Y_t_* at time *t*, is said to be autoregressive if there is a regression (a nonzero regression) of *Y_t_* on *Y_t −_*_1_, for all values *t*. This concept applies to stationary time series, meaning those having probability distributions that are time-invariant (see Ref. 29, Chapter 18). More generally, a time series is autoregressive with order *p* if there is a regression of *Y_t_* on the values *Y_t −_*_1_, *Y_t −_*_2_,…, *Y_t − p_*, going back in the past *p* time steps. Autoregression is the most straightforward way to model dependence across time. Vector autoregressive (VAR) models extend this concept to multiple time series.

With this in mind, a time series of values *X_t_* is Granger causal of a time series of values *Y_t_* if the regression of *Y_t_* on the values *Y_t −_*_1_, *Y_t −_*_2_,…, *Y_t − p_* together with the values *X_t −_*_1_, *X_t −_*_2_,…, *X_t − p_* is stronger than the regression of *Y_t_* on *Y_t − _*_1_, *Y_t − _*_2_,…, *Y_t − p_* alone. This verbal summary is meant to provide a succinct description of the concept of theoretical stationary time series; there are many important details in implementation and practice, and cautionary concerns about the application of Granger causality appear under time-varying dynamics, below. In addition, Granger causality is something of a misnomer because, as we discuss in *Causality*, it is rarely causal in the usual scientific sense.

There are extensions of Granger causality based on directed information (72–74), as well as extensions that apply to spike trains (75–77). Furthermore, the autoregressive structure may be replaced with more flexible nonlinear relationships, including those generated by neural networks (78, 79).

### Latent Dynamic Models

In latent dynamic models, a latent variable (typically a vector) takes values often known as states, which evolve dynamically, across time. The latent state variable represents theoretical quantities of interest, and its path across time is estimated from observations, often with the goal of prediction. The framework for observation at time *t* in terms of states and noise at time *t* may be designated in the general form,

(*2*) $${\text{observation}}_{t}\overset{\text{combines}}{\equiv }\;{\text{state}}_{t}\overset{\text{with}}{+}{\text{observation noise}}_{t},{\text{state}}_{t}\text{ evolves according to a stochastic process}$$

where “combines … with” signifies that states are being combined with noise on the right-hand side to produce the left-hand side. When “combines … with” is made precise in mathematical equations, the first line of *Eq. 2* defines the way states determine observations (aside from noise) while the second line defines how the states evolve across time (see *Stochastic Processes*) within what is usually called a “state space.” The first line is often called the observation model while the second line becomes the state model. Important special cases are linear dynamical system models (LDS models, also known as linear state-space models), latent Gaussian process models, and the many generalizations of these two special cases; we give a few pointers to relevant literature in three successive subsections (see also Ref. 23).

In theory, stochastic processes can be used to formulate continuous-time state-space models but practical implementations revert to discrete time. Although this may make the distinction between discrete and continuous time seem unimportant, some formulations have origins, and, thus, intuitions and useful results, grounded in continuous time. Models that are inherently discrete, and typically have additional structural simplifications, are usually called state-space models. When “combines … with” involves linear models (and, for many purposes, Gaussian noise), the modifier “linear” may be included but, because this special case is so common, it is frequently taken as a default with “linear” omitted.

**Figure 8.**
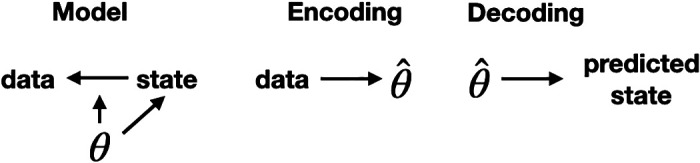
Encoding and decoding. The observation model, the first line in *Eq. 2*, is depicted in the left-most diagram by the arrow leading from state to data. Both the observation model and the state model (in the second line of *Eq. 2*) depend on parameters, collectively written here as a vector θ. Encoding uses the data to produce an estimate $\hat{\theta }$ of θ. Decoding uses the estimated model parameter vector to produce an estimate, or prediction, of the state vector. In typical brain-computer interface applications based on motor cortical activity, the state vector includes kinematic variables such as velocity that control a robot arm or a cursor on a screen.

In neuroscience, state estimation is often known as “decoding,” with “encoding” referring to the process of estimating unknown parameters in the model, such as those appearing in the linear transformations and the noise distributions, see Fig. 8. The first application to a decoding problem was the study by Brown et al. (80), who showed how the latent dynamical modeling framework could predict navigational paths based on multiple spike trains recorded from hippocampal place cells. Brain-computer interface applications for hand movement adapted this general scheme (81, 82).

In applied mathematics, the term “state space” is synonymous with “phase space,” where it refers to the possible configurations of a *dynamical system* that evolves in continuous time, moving from a state at time *t* to another state at time *t′*, according to differential equations, without being stochastic as it is in *Eq. 2*. Much of theoretical neuroscience involves dynamical systems in this sense (e.g., Ref. 83). Dynamical systems have played a prominent role in theories of motor control (84, 85) and in data-analytic procedures aimed at understanding the neural control of movement (86), with the concepts and techniques being seen as applicable to many other neural circuits (42, 43). The motivation for latent dynamic models often leans on rough physical intuition, at least in the sense that relatively simple models can provide insight into dynamics. The term “dynamical system,” however, is often used in statistics and signal processing (87) to describe the noisy evolution of states in *Eq. 2*, and almost always refers to statistical state-space models, discussed very briefly in the following subsection; linear state-space models are often called linear dynamical system (or LDS) models. One concept in applied mathematics of dynamical systems that might profitably receive more attention in neuroscience is the Koopman operator (88, 89), which extends greatly a method known as dynamic mode decomposition (90).

#### State-space models.

In state-space models, the probability that a latent variable will be in a specific state at a particular time is typically restricted to depend on the most recently occurring state but not on earlier states (which is known as a Markov assumption). When the form of combination in *Eq. 2* involves a linear transformation of the state to the observations with additive noise in the first line (the observation model), and the second line (the state model) has the form

$${\text{state}}_{t}\overset{\text{combines}}{\equiv }\;{\text{state}}_{t-1}\overset{\text{with}}{+}{\text{state noise}}_{t},$$

where the combination involves an autoregressive model for the states and when, in addition, the noise is Gaussian, the resulting procedure that produces state estimates is known as the Kalman filter (e.g., Section 16.2 of Ref. 29). In their application to place cell decoding, Brown et al. (80) used a state-space model to accommodate Poisson spiking based on a generalized linear model (GLM, Ref. 29, Chapter 14), rather than the linear Gaussian form. For general discussions, see the book by Chen (91) and the article by Eden et al. (92). Semedo et al. (93) used a state-space model to identify the strength of cross-area interactions. By studying cross-area interactions on the level of the latent states, their method provided a succinct description of the interactions between visual areas V1 and V2 and described a time-structure distinction between within-area and cross-area interactions.

#### Gaussian processes.

A Gaussian process (GP) is a stochastic process in which all the multivariate joint distributions are Gaussian. This requires specification of the covariance of variables $X_{t_{1}}$ and $X_{t_{2}}$ for every pair of time points *t*_1_ and *t*_2_. To make analysis tractable, GP technology has emphasized particular forms of covariance, which restrict the appearance, especially the smoothness, of the resulting trajectories. Yu et al. (94) replaced the static latent factors in ordinary factor analysis (depicted in Fig. 5*A*) with Gaussian processes (Fig. 5*C*) and showed how the resulting Gaussian process factor analysis (GPFA) could capture important features of multiple spike train behavior during movement, especially through visualization of latent trajectories across time. Although many possibilities for covariance functions have been discussed in the statistics and machine learning literature (Ref. 95, Chapter 18), the most common of these contains a single parameter that determines the smoothness of the trajectory (a time constant for the covariance decay). The implementation by Yu et al. (94) allowed each latent factor to have its own smoothing parameter and thereby provided multiple timescales for population representations. The latent variable structure of GPFA allows it to be considered a firing-rate model, and it reduces Poisson-like noise (see *Theoretical firing rates as latent variables*) similarly to, though differently than, Poisson-based or point process models.

Gokcen et al. (96) extended GPFA to multiarea cases by using a Gaussian process that had both area-specific and interacting components, with the interacting component including a delay variable to represent the time lag between areas. Applied to simultaneous recordings from visual areas V1 and V2, their method found bidirectional, asymmetric, and selective interactions.

Bong et al. (59 and 65) used latent Gaussian processes as in GPFA, except unlike the usual Gaussian processes that restrict the covariance structure, their approach allowed general (unrestricted) autocorrelations and cross-correlations, within a fixed window (time lags up to 100 ms in their applications). Their primary model, depicted in Fig. 5*D*, is a generalization of pCCA to time series (in a precise sense they formulated and proved). The model was thereby very flexible but it created a large number of parameters. They, therefore, developed an approach to estimation and inference (confidence intervals and significance tests) based on known strategies for high-dimensional problems (see the discussion under *High dimensionality* in the *Defining Neural Populations* section), and they applied the methods to find time-varying beta amplitude coupling between PFC and V4 during a visual memory task.

#### Generalizations.

The linear-Gaussian (or LDS) model and accompanying methodology have been extended widely, demonstrating the depth and breadth of the latent dynamical modeling framework (Ref. 95, Chapter 29). One of the basic extensions is to allow the system to evolve differently, at different times, according to one of several distinct linear dynamic regimes, leading to switching dynamical models often known as switching LDS (SLDS) models. In describing these, let us call the states in *Eq. 2* instead *latent drivers* (they drive the population activity), and then let us refer to the different dynamic regimes as being in alternative states. With this terminology, SLDS models are called *recurrent* (rSLDS) if the latent drivers influence the probability of being in a particular state. A series of papers have developed these for spiking data. Glaser et al. (97) included population-specific drivers and state probabilities while allowing the probabilities of staying within states to be different from the probabilities of moving to new states. The authors documented ways the dynamics within and between M1 and the dorsal premotor area (PMd) changed as the task evolved from a delay period to a movement period during a reaching task.

**Figure 9.**
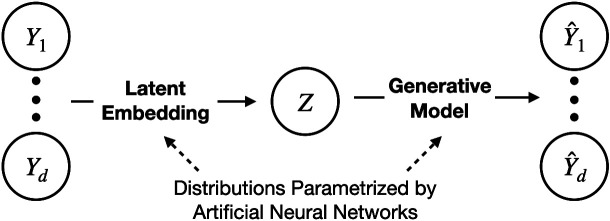
Variational AutoEncoder (VAE) model. This model combines the encoding and decoding of Fig. 8 and uses artificial neural networks for each of the two parts. The labels “latent embedding” and “generative model” are terms used in the literature, but refer to the same processes and have the same general structure as encoding and decoding in state-space models. The “variational” modifier refers to variational inference, which uses a simplification to approximate the Bayesian calculations used for encoding in standard state-space models, such as linear dynamical systems.

A further extension is to allow either the mapping from latent drivers to observations or the evolution of the latent drivers (or both) to involve neural network models. Gao et al. (98) showed how multiple spike trains could be analyzed using a feed-forward neural network model to map from latent drivers to observations, where the states evolved linearly. In addition, Pandarinath et al. (99) used a recurrent neural network to replace the Gaussian processes in GPFA, and artificial neural networks for encoding and decoding in an architecture known as a Variational Auto Encoder (VAE, see Fig. 9), which improved predictive performance for single populations of spiking motor cortical neurons. Karniol-Tambour et al. (100) then extended the multipopulation approach to rSLDS models [as discussed by Glaser et al. (97)] to incorporate a recurrent neural network that represented the evolution of latent drivers (as opposed to the linear dynamics in rSLDS). Analyzing calcium image data, they described feedforward drive from V1 to V2 and posterior parietal cortex (PPC) and feedback from PPC to V1 and V2. Such an approach would apply to spike trains with a relatively minor modification to the observation distributions.

Neural networks may be especially valuable when they can be used in hybrid models that incorporate components involving interpretable parameters together with components for mapping functions (represented by neural networks) that do not require interpretation. The neural networks would then involve high-dimensional nuisance parameters in the sense of our preliminary remarks in *Latent drivers and hierarchical interactions*.

### Frequency Analysis

**Figure 10.**
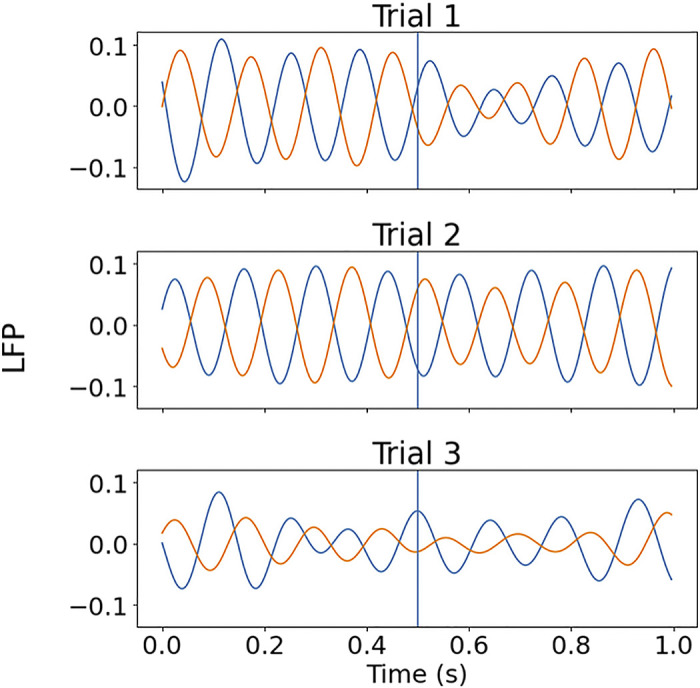
Coupling of phases across trials. Orange and blue lines denote two different theta-band filtered local field potential (LFP) signals, for three trials. The vertical blue line illustrates a potential time at which the phase is measured across the trials. There is a consistent pattern of phase offset between the two signals, with one signal offset from the other by about half a cycle, subject to some variation, but the phases at any specific trial time point vary across trials. The filtered LFPs also display amplitude variation, within and across trials.

Interest in neural oscillations has a long history (e.g., see Ref. 101), and the potential physiological relevance of oscillatory cross-area interactions has been discussed in many places (102, 103). Much of the literature concerns EEG and MEG recordings which, while methodologically different, produce data structured and analyzed similarly to LFP data. The analytical starting point is a decomposition of oscillatory signals into idealized frequency components. In the oldest and still-dominant case of Fourier analysis, the signal is decomposed into sinusoidal curves at many frequencies, each of which has both an amplitude and a phase (see Ref. 104, Chapter 13 and Ref. 29, Chapter 18). Thus, a pair of signals from two areas can exhibit correlated behavior (across trials, see Fig. 10), in various ways, involving phases or amplitudes (or both) at a given frequency, or in combinations of frequencies.

Mathematically, amplitude and phase may be considered together as defining a complex number, and a kind of complex-valued coupling leads to the widely-used measure known as *coherence*. Coherence fits naturally into a now-classical theoretical framework for understanding the coupling of oscillatory components in signals (Ref. 105, Section 4.6) and is usually said to be the magnitude of the correlation between two signals at a given frequency. Although there is a sound mathematical basis for that verbal description (the Cramér-Khinchin decomposition), it ignores the distinction between the familiar real-valued Pearson correlation and the correlation being referenced, which is a complex-valued correlation of complex random variables. Although complex-valued correlation is, in some respects, similar to the more familiar real-valued correlation, it is different; we return to it below. Coherence depends not only on phase coupling but also on amplitude variation, which can sometimes make interpretation challenging. An alternative phase coupling assessment, known as Phase Locking Value (PLV), ignores amplitude and, under certain commonly-found circumstances, may be considered an analog of Pearson correlation that applies to angles. When the amplitudes are nearly constant, coherence and PLV behave similarly, but the frequent presence of amplitude fluctuation has led some analysts to prefer PLV. For a comparative summary, and references, see Urban et al. (68).

In the same sense that coherence is analogous to correlation, partial coherence (e.g., see Ref. 106) is analogous to partial correlation (which was illustrated in Fig. 2*C*). The torus graphs developed by Klein et al. (67) provided a multivariate generalization of PLV and, thus, as we mentioned in *Graphical models*, the framework also provided measures analogous to partial correlation. Klein et al. (67) discussed an important special case of torus graphs, which had been suggested earlier by Cadieu and Koepsell (107), based on coupled oscillators. Lead-lag structures in the frequency domain can be identified using partial directed coherence (108) and spectral causality (109), the latter being a frequency-domain version of Granger causality estimated through VAR models (see *Autoregressive Models and Granger Causality*).

Cross-frequency coupling of theta and gamma oscillations in LFPs recorded from the hippocampus was described by Belluscio et al. (110), and this is often considered a form of phase-amplitude coupling (111). Additional statistical references can be found in the study by Ombao and Pinto (112). General statistical considerations concerning cross-frequency coupling were discussed by Savolainen (113). Amplitude-amplitude coupling has also been observed (114). Siegel et al. (102) review the different categories of interactions between phase and amplitude and their interpretation in the context of neural data.

Urban et al. (68) used the important observation that coherence can be obtained as the magnitude of the complex-valued correlation between two signals after narrow band-pass filtering [spelled out in detail by Ombao and Van Bellegem (115)], to develop a complex-valued latent variable model. Because the model is based on the complex normal distribution, it provides a framework for identifying probabilistic graphical models (as described in *Dimensionality and multivariate models*). Under that model, when maximum likelihood estimation of the latent correlation matrix is applied to narrowly band-pass filtered data, each correlation becomes a latent coherence, while each partial correlation becomes a latent partial coherence (the method is a generalization of pCCA to the complex domain). One of the data analytical results is displayed in Fig. 7*B*.

The time evolution of frequency coupling can be studied with several methods, including time windowing (e.g., Ref. 29, Section 18.3.7) or wavelets (e.g., Ref. 104, Chapters 12 and 13). Fiecas and Ombao (116) used time-localized spectra (periodograms) to identify time-varying interactions in LFPs recorded from hippocampus and the nucleus accumbens. As mentioned under *Gaussian processes* in the *Latent Dynamic Models* section, Bong et al. (59 and 65) and Liu et al. (Liu Z, Bong H, Ren Z, Smith M, Kass RE, unpublished observations) developed methods for identifying time-varying amplitude-amplitude interactions.

When a neural spike train is driven by an oscillation, unless that oscillation is very powerful, recovering it is challenging statistically (117). Frequency analysis of point processes requires care. Investigators sometimes apply standard frequency analysis techniques to smoothed spike trains, but this can lead to erroneous conclusions (Refs. 29 and 118 Section 19.3.7). Related cautions apply to spike-field coherence (119).

### Point Process Models

A point process model describes spike trains by assuming at most one spike can occur at any point in time, and the probability of spiking is governed by a firing rate function (in continuous time). In practice, spikes are recorded in small time bins, and if the bins are small enough that at most one spike can occur in each bin, the point process defines a bin-specific probability that a spike will occur and the spike count (0 or 1) in each bin may be considered to follow a Poisson distribution. Because of possible statistical dependencies across bins, the spike counts in larger time bins need not be Poisson, and statistical point process models can mimic a wide variety of spiking behavior, including the Poisson-like yet discernibly non-Poisson behavior of observed spike trains that are commonly observed in vivo in the cortex (120–122).

Formally, a point process statistical model for a spike train involves two things: *1*) a simple, universal formula for the probability density of the spike train in terms of the theoretical firing rate and *2*) a specification of the way the theoretical firing rate depends on relevant variables. The variables determining the theoretical firing rate could include measurements characterizing the stimulus, or behavior, or the past spiking of the neuron itself or other neurons, and it often becomes a latent variable (as discussed in *Latent variables*). Point process models are typically implemented using standard generalized regression methods (Ref. 29, Chapter 19), where possibly nonlinear effects (e.g., due to a stimulus or the spiking of other neurons) are represented using basis functions (such as splines). Because the framework is intuitive, mathematically rigorous, and able to take advantage of modern statistical methods, point processes have long been considered the natural statistical approach to modeling spike trains (123–125). Increased knowledge, including documentation of their close relationship to integrate-and-fire models, has only made the theoretical case for their use more compelling (Ref. 26, Section 2.4 and Ref. 122), and there is substantial literature on point process methods for spike train analysis, with historical markers including Kass and Ventura (126), Truccolo et al. (127), and Pillow et al. (128). There are some important implementation issues including the degree of smoothness assumed in the firing rate function and the precise form of non-Poisson spiking. A detailed discussion of the similar but different alternative modeling assumptions used by Kass and Ventura (126) and Pillow et al. (128) were given by Chen et al. (129). Many additional references may be found in studies by Keeley et al. (23) and Meyer et al. (130). Point process models also form a statistical foundation for analysis of precise spike timing effects, as discussed in Section 3.4.2 of Ref. 26 and references therein, though these have been applied mainly within populations.

Although point processes may have a strong statistical motivation, they are models for spike counts in infinitesimally small time bins, usually approximated by time bins having 1 ms duration, and for some purposes it is entirely adequate to consider broader time windows. As a consequence, many useful analyses have applied simpler methods. Similarly, while a version of GPFA that works well for very small time bins has been articulated (131, 132), the original formulation, which uses Gaussian distributions rather than assuming the data to be binary, is less cumbersome. Point processes become a method of choice when it is advantageous to model the evolution of firing rate functions with millisecond precision.

**Figure 11.**
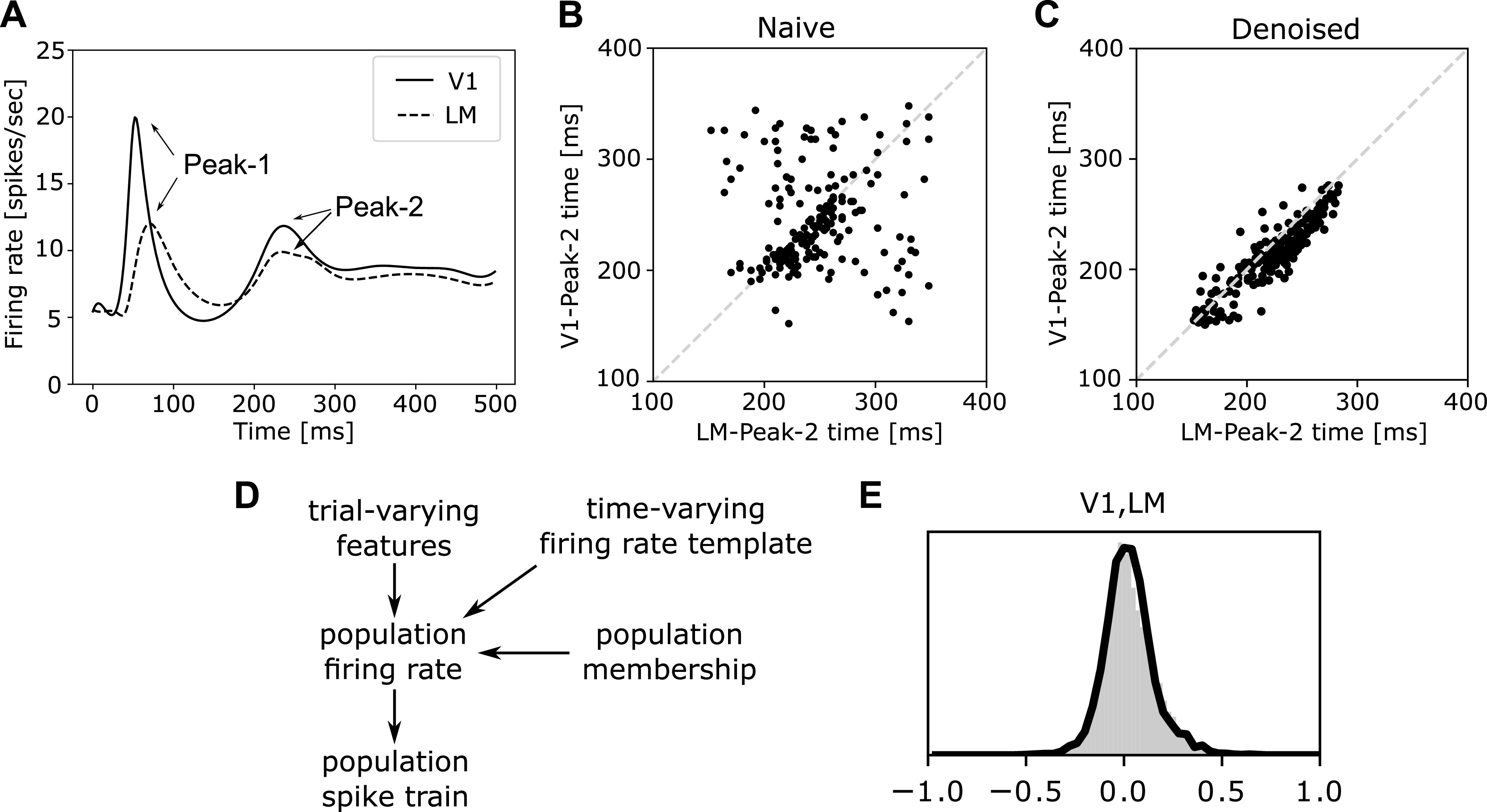
Cross-area coupling of population peak firing rate timing. *A*: smoothed population peristimulus time histograms (PSTHs) in response to full-field drifting gratings for mouse visual areas V1 and LM. The two population PSTHs have similar features: the first peak (Peak-1) appears early, soon after stimulus onset while the second peak (Peak-2) appears much later. *B*: plot of the time of Peak-2 in V1 against the time of Peak-2 in LM based on smoothing the two PSTHs on a trial-by-trial basis (näive method). Each data point represents the pair of peak times on a particular trial. There is no visible relationship (and no statistically significant correlation). *C*: results plotted as in *B* except now based on the model of Ref. 27. In this plot the correlation is strong (0.87, standard error 0.025), with Peak-2 in LM tending to occur later than Peak-2 in V1. *D*: schematic of the statistical model, discussed in *Point process models*. *E*: spike count correlation histogram when one neuron is in V1 and the other neuron is in LM, which is mildly skewed toward positive correlations. The model prediction (dark line) fits the histogram well, even though the only sources of correlation in the model are the trial-by-trial shifts in the timing of the population peaks and trial-by-trial changes in the overall firing rate of each population; in generating the model fit the neurons were assumed to fire independently, aside from their shared population-level trial-to-trial variability. Figure modified from Ref. 27.

An example is the method by Chen et al. (27) which, as illustrated in Fig. 11, focused on the time of maximal firing rate in response to a stimulus. Because, by definition, many spikes occur near the time of the maximal firing rate, the time itself will typically be well-determined, statistically. Particularly when compared with the relatively small spike count correlations found in cortical recordings (133, 134), the trial-to-trial correlation of population Peak-2 times across areas V1 and LM, of 0.87 ± 0.025, plotted in Fig. 11*C*, is striking. Those authors also found the time lag from Peak-2 in V1 to Peak-2 in LM to be, on average, 9.6 ms, with a standard deviation across trials less than 2 ms and contrasted this relatively small trial-to-trial variation with the large trial-to-trial variation in Peak-2 timing itself for both visual areas, where the standard deviations across trials for both areas were roughly 30 ms. Furthermore, even though their statistical model accounted for trial-to-trial variation only at the population level, when Chen et al. assumed that, beyond the population-level variation, all neurons were firing independently of each other, as shown in Fig. 11*E*, the model could match closely the observed spike count correlation histogram across pairs of neurons.

In *Dimensionality and Multivariate Models*, we noted that functional connectivity between two or more populations can be analyzed either by modeling directly the coupling of activity across the populations or by modeling the predictability of each recording from the many recordings across the other populations; the latter could be called a regression approach, as in Fig. 3*C*. Both strategies have been applied to point process analysis of spike trains. The regression approach has been used by Hart and Huk (135), Stevenson et al. (136), and Zoltowski and Pillow (137). For example, Hart and Huk (135) used point process regression models to compare the strengths of interactions within and between LIP and FEF, and to show that the strengths of interactions changed from a fixation period to a delay period in an oculomotor task. At the population level, the possibility of applying point processes that include latent variables in the form of state models or Gaussian processes has been discussed by Keeley et al. (23), based on successful implementations of such models within a single population (e.g., Ref. 138).

Chen et al. (27) attacked population coupling by selecting neurons within each population that had similar theoretical firing rate variation across time, pooling together the selected spike trains within each population (i.e., merging all the spike times) to define a population spike train, and then modeling the covariation of particular features of the population firing rate functions, including the times of peak firing rate as shown in Fig. 11*A*. The major components of the model appear in Fig. 11*D*. Importantly, the neurons representing each population (“population membership”) were selected separately for each experimental condition (by incorporating model-based clustering into their Bayesian procedure). The population firing rate function combined a condition-specific time-varying template (for the overall shape) with trial-varying features, which were the two peak firing rate times and a gain constant, all of which are likely to be relatively well-determined by the data and thus well-estimated. The population firing rate function for a given trial (in a given brain area) then produced a population spike train by pooling together the spikes produced by all the neurons in that condition-specific population. As we mentioned earlier, the method also corrects for attenuation of correlation, illustrated in Fig. 1. Chen et al. (27) examined covariation among the three visual areas V1, LM, and AL to find results like those in Fig. 2*C*.

As yet unpublished work by Olarinre, Siegle, and Kass used a simpler approach than that of Chen et al. (27), corroborating results while verifying the importance of modeling (and thereby greatly reducing) the Poisson-like noise (discussed in *Theoretical firing rates as latent variables*) and fitting population firing rate functions separately for each experimental condition based on condition-specific neural populations. They used the simpler approach to analyze the mouse-to-mouse variation of peak times and the correlation of peak times across seven brain areas. Additional, related, unpublished work by Xin, Siegle, and Kass demonstrates ways in which population firing rate coupling models can be much more accurate than individual neuron models for identifying cross-population interactions, and they use population firing rate models to document strong diminution of particular interactions across visual areas during locomotion.

## IMPORTANT ISSUES

Every statistical model has assumptions, and every analytic method highlights some features of the data while ignoring others. We have not listed the many caveats that accompany each approach. In this section we make cautionary remarks, confining them to a few points that may be easily overlooked or ignored.

### Defining Neural Populations

#### Neural population diversity.

In spike train analysis, the sampled neurons are inhomogeneous, with both excitatory and inhibitory cells varying in properties, genetic makeup, location (including depth in the cortex), and function (4). Typically, only task-relevant neurons, having firing rates that increase substantially in response to a stimulus or behavior, are used in analyses, but among these considerable diversity remains (139). In population-level analysis, the constitution of a relevant neural population is often left vague, the apparent assumption being that the haphazardly sampled neurons do a reasonably good job of capturing important effects. In some cases, however, populations could involve only a small percentage of neurons within some designated area; they could be condition-specific or behavior-specific or state-dependent, and they could involve particular classes of neurons. For example, Chen et al. (27) identified relatively small condition-specific groups of neurons that participated in cross-population coupling (in the sense those authors were examining). The strong correlations observed by Chen et al. (27), illustrated in Fig. 11*C*, suggest this kind of selectivity can be an effective analytic strategy and could inform conceptions of population interaction.

Local field potentials also arise from many diverse sources, which may have very different activation patterns. One approach to disentangling the signals is through current source density localization (16, 140), methods for which continue to be developed, sometimes with an eye toward cross-population coupling. Klein et al. (141) used Gaussian process current source densities to map alpha-band phase coupling between lateral and medial subareas of the primary auditory cortex following tone presentations; the results could not be found from direct application of phase coupling methods to the LFPs themselves.

#### High dimensionality.

While large numbers of electrodes create great opportunities to attack complexities in cross-population coupling, they also create computational challenges, which a small body of work has addressed (e.g., Ref. 142; see also Ref. 23). More fundamentally, it is hard to fit accurately the large numbers of parameters involved in high-dimensional statistical models. In practice, quantities of data that seem very large in raw size (number of bytes) may actually be small when it comes to answering, reliably, a question being posed because a new recording capability has become available. This is the problem of granularity: as new scientific queries are generated by refining old ones, the number of possible results, which are typically defined by combinations of variables (e.g., identifying which neurons across all areas are interacting), grows rapidly, so that the amount of data bearing directly on a particular query is greatly reduced. This makes it difficult or impossible to gain knowledge without somehow limiting the possibilities under consideration. Thus, every valuable statistical approach must, explicitly or implicitly, introduce some assumption that makes the problem tractable.

A class of approaches for solving certain high-dimensional problems comes under the heading of *regularization* (Ref. 44, Sections 11.3–11.4). This terminology comes from situations involving variables that are mathematically redundant, or nearly so, in the sense of our introductory comments in *Dimensionality and Multivariate Models*. One popular, and often productive statistical assumption is known as sparsity, according to which there are comparatively few large effects (such as those quantifying interactions), and the small effects are not important. In settings such as linear regression and generalized regression, this leads to a computationally efficient and well-studied method known as L1 regularization, often called Lasso, which reduces the size of parameter estimates (it “shrinks” them) and sets the smallest estimates to zero. L1 regularization has been used to analyze multiple simultaneously-recorded spike trains where a given neuron’s spiking behavior could be functionally related to the spiking behavior of large numbers of other neurons (e.g., Ref. 143).

In neural applications, it often happens that there are not a small number of large effects (e.g., neurons functionally connected to each other) but rather a large number of small effects. This calls into question the assumption of sparsity. In addition, statistical inference with L1 regularization can be challenging. Two ideas have been used to improve the situation. First, it is known that Lasso is generally better at prediction (predicting the firing rate of one neuron based on firing patterns of many others) than model selection (finding correctly the set of neurons that have strong interactions with a given response neuron): intuitively, a good set of “wrong” variables can predict nearly as well as the right ones (because the wrong variables are, collectively, correlated with the right ones); see the discussion by Bühlmann (144). Bong et al. (65) applied general results that take advantage of this fact (145, 146) to identify time-varying beta-oscillation amplitude coupling of PFC and V4 during a memory task from LFPs recorded from Utah arrays.

Often, special-purpose methods can improve on generic Lasso-based approaches by incorporating scientifically reasonable assumptions. This was the strategy of Vinci et al. (66), discussed in *Graphical Models*, where the usual L1 regularization for variance matrix estimation known as the graphical Lasso was modified by allowing the shrinkage of partial correlation for each pair of firing rates to depend on the distance between the two neurons and their tuning curve correlation. This produced the striking improvement in performance shown in Fig. 6*A*.

### Time-Varying Dynamics

Traditionally, interactions across brain areas have been depicted as static network snapshots. In reality, brain networks are dynamic and constantly changing. In many situations, the changes across time are of central interest. Tracking such changes is the primary motivation not only for the peristimulus time histogram (PSTH), but also for comparatively recent methods such as GPFA.

A particular challenge, affecting both spike trains and LFPs, is large fluctuations driven by stimulus or behavior, often called evoked or transient (or phasic) responses, which are clearly visible in typical PSTHs and trial-averaged LFPs. Such pulsatile fluctuations are difficult to reconcile with the statistical assumption of stationarity (time invariance), which is fundamental to common time series procedures. Repeated trials can provide the information needed to disentangle transient fluctuations from the kind of steady-state variation standard time series methods are built to handle. In GPFA, multiple processes with different effective timescales can often accommodate transient fluctuations while also capturing background variation. However, application of Granger causality requires care, and must be modified or abandoned in the presence of transient activity (69, 73, 147, 148). The same is true of directed information.

At the opposite end of the spectrum, a variation that is slow relative to the length of the trial creates what is known as long-range dependence, which also causes problems. In time series models, the dependence between observations at different points in time, often measured by their correlation, which in this context is called autocorrelation, tends to get smaller as the time between the two observations increases. When the autocorrelation function decays exponentially fast, the dependence is called “short range,” and when it instead decays slowly the time series exhibits “long-range dependence.” Even though long-range dependence can satisfy stationarity, when long-range dependence is present typical time series procedures will behave poorly, producing effects that are similar to nonstationarity (149). If, for example, there are trial-dependent waves of activity that elevate the average activity on some trials compared with others, observations near the end of a trial will tend to be correlated with observations near the beginning of a trial, thus exhibiting long-range dependence. When autoregressive models are fit to data, the best-fitting model may be the one having the greatest number of autoregressive terms, meaning that it allows dependence on values at a maximal distance in the past; this would be an indication of likely long-range dependence or nonstationary behavior.

A conceptual complication, which often produces some confusion, involves the characterization of signals exhibiting long-range dependence as “1/*f* noise,” by which it is meant that the spectral density (the power spectrum), written as a function of frequency *f*, decreases either exactly or approximately as 1/*f*, though the terminology often refers to decreases that have a more general power law form 1/*f*^ α^, with α not necessarily close to 1. The exact or approximate form 1/*f* contrasts with spectra that decrease at least as rapidly as 1/*f*^2^, which are seen in standard statistical models exhibiting short-range dependence, such as autoregressive models and the most commonly-applied Gaussian process models, all of which would be considered “well-behaved.”

**Figure 12.**
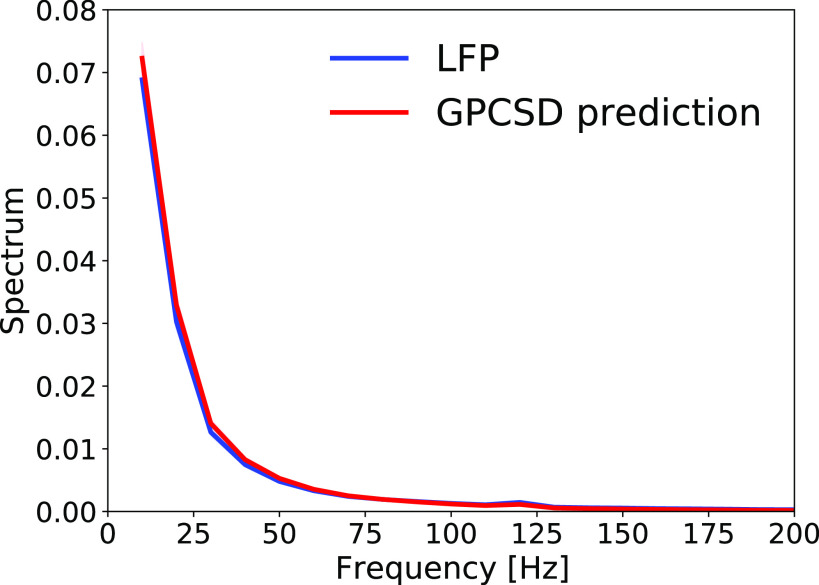
Local field potential (LFP) spectra. A comparison of the spectrum of an LFP from primate primary auditory cortex (A1) and the spectrum of the fitted LFP using the method of Ref. 141, called Gaussian Process Current-Source Density analysis (GPCSD). The spectrum of the LFP was computed after first subtracting the deterministic transient component (blue); the remaining two-component Gaussian process model (red) fits the spectrum very well.

The confusion comes from a few sources. First, in some articles, the terminology “1/*f*” may refer to any or all of three statistically different cases: exactly 1/*f*, approximately 1/*f*, or more general power law cases (including 1/*f*^2^). Second, when a time series appears to exhibit long-range dependence it is difficult, statistically, to determine the precise decay rate of the spectral density. Third, there are several phenomena that may or may not accompany and explain long-range dependence, including scale invariance, chaotic evolution, and self-organized criticality. More specifically, there are many ways to generate time series having spectra that decrease as 1/*f*^ α^, including the special case α = 1. In particular, beginning with a Poisson process that produces decaying voltage pulses, as in the usual conception of synaptic input, various realistic considerations (the precise form of decay; a combination of multiple independent processes; time-varying intensities) can lead to exact or approximate 1/*f*^ α^ spectra, with the value of α depending on the precise specification (150, 151). However, in practice, a very small number of short-range dependent processes, such as three processes, can produce spectra that are statistically (and visually) indistinguishable from 1/*f* (152). For example, as shown in Fig. 12, Klein et al. (141) obtained very good fits to multielectrode LFP recordings having spectra that appeared to exhibit long-range dependence when they assumed the current source density (which produced the LFPs) had a 3-component form they labeled “transient + slow + fast,” where the transient component was a deterministic function (representing the evoked response) and the two other components were well-behaved Gaussian processes with two different time scales for “slow” and “fast” (which were learned from the data) and the fast component could accommodate alpha-band oscillations. They then used the current source densities to analyze cross-population coupling.

The two standard remedies for long-range dependence, which are equivalent conceptually but may differ in detail, are either to model it, as in the study by Klein et al. (141), or to fit the slow trends and then analyze residuals after trend removal (e.g., Ref. 29, pp. 528–529). When it is safe to assume stationarity, with short-range dependence, LFPs can be decomposed into frequency components. Instead of stationarity across the entire trial, local stationarity is often assumed, effectively meaning that stationarity holds within smaller windows of time; references were given in *Frequency Analysis*. Ombao et al. (153) defined an efficient procedure for breaking trials into a relatively small number of blocks within which stationarity could be assumed and Hefny et al. (154) extended that model and applied it to multiple LFPs.

### Trial-to-Trial Variability and Poisson-like Noise

Substantial trial-to-trial variation is visible in both spike trains (where it is typically seen in raster plots of in vivo cortical data) and LFPs (where very large numbers of trials are usually averaged to see effects). Trial-to-trial variation is often called “noise,” especially in the context of spike count correlation (133, 134, 155). A source of variability is labeled “noise” to suggest it is either not understood or thought to be irrelevant for some limited purpose, and the label may not be appropriate in a different context (we might say, “Today’s noise is tomorrow’s signal”). In particular, calling it noise is not intended to imply that trial-to-trial variation is uninteresting: when neural activity covaries across trials in two areas, something has produced it. Covariation indicates shared participation in circuits, at least in the sense that activity in one area affects activity in the other; or the two areas are driven to some identifiable extent by inputs not too far upstream that they have in common; or both.

There are at least four categories of sources for the variation in neural activity across trials. First, there may be subtle discrepancies in repetitions of the experimental paradigm, including the measurement process, or the manner in which the subject participates. During the presentation of a visual stimulus, for example, even small deviations in foveal fixation might change responses of retinal ganglion cells, and thus the whole visual system. Second, endogenous states affecting neural activity, such as those associated with arousal or attention, may vary across trials. These first two sources may account for the large trial-to-trial variation in the mapping between experimental time and neural response seen in Fig. 11 (see the legend for Fig. 11*C*). The third source is stochastic behavior in the flow of ions and neurotransmitters into and out of each neuron, and the fourth, at least in cortex, is the apparent widespread, low-level balanced network activity (see Ref. 156 and references therein), which has been hypothesized to benefit transmission of information (120, 157) and may arise from chaotic behavior of neurons that behave like sparsely connected leaky integrators (158). The latter two sources, and perhaps other sources, produce what we called “Poisson-like” variation in *Point process models*, meaning that spiking patterns are similar to those produced by a Poisson process; although close examination typically reveals them to be noticeably different, neural spiking patterns are often not far different from patterns produced by Poisson processes (Ref. 29, Chapter 19). Furthermore, point process models that can accommodate deviations from Poisson variation are similar mathematically to Poisson process models, which constitute an especially tractable special case. Thus, Poisson-like noise will tend to produce a trial-to-trial variation that is consistent with that described by point process models.

### Causality

The methods we have discussed are nearly always used in studies that are not designed to find causal influences of one area on another. This is why we said, earlier, that Granger causality is something of a misnomer. Granger causality is a very natural and useful statistical tool, but it does not assess causality in the usual scientific sense; it is instead predictive. Experimental investigators know that correlation does not imply causality: in general, an observed correlation between variables *X* and *Y* could be due to a confounding variable *W*, in which case if *W* were held fixed, changing the value of *X* would not change the value of *Y*. Statistical evidence that *X* causes *Y* can be gained only when confounding is highly improbable, such as when the values of *X* are assigned with a randomized procedure. Thus, in the absence of reasons to think confounding is highly improbable, Granger causality is predictive but does not provide evidence of causality.

A different dichotomy that helps to clarify discussions of interacting neural populations contrasts causes of effects with effects of causes (159). The former aims to label the goal of reasoning from observations back to causal influences, while the latter refers to situations in which outcomes (effects) of differing experimental conditions (causal manipulations) are observed. Not only is the latter at the heart of experimental science, but it is also the presumptive situation in which causal statistical reasoning can be valid. The field of statistics has developed a workable definition of causal effects and a body of methods for establishing them. An elementary overview is given in the book by Rosenbaum (160); additional details may be found in the work cited there and in the book by Hernán and Robins (161).

What should it mean to say that neural activity in one population, represented by a variable *X*, is a cause of activity in another population, represented by a variable *Y*? Causal statements use imagined situations: a comparison is made between values of *Y* (or probability distributions of *Y*) when values of *X* change (e.g., a population-average firing rate goes from 2 Hz to 25 Hz), while all of the inputs to the neurons whose activity is represented by *Y* (those inputs that are not themselves driven by *X*) remain the same. Because it is not possible to observe neural activity *Y* under “exactly the same brain activity configurations” for two different values of neural activity *X*, a comparison of them involves *potential* outcomes, which are also called “counterfactual.” Statistical causal analysis thus defines causal effects in terms of potential outcomes, and under special circumstances characterizing the design of a study (including but not limited to randomized controlled trials) it is possible to obtain causal statements (160, 161). In the absence of causal manipulation, when confounding variables having unknown effects could be present, statistical analysis cannot discover causes of effects while also demonstrating conclusively that they are causal (162).

One of the difficulties, mentioned in *Graphical Models*, arises even under relatively strong qualifying assumptions. Suppose, for example, that *X*_1_, *X*_2_, and *X*_3_ represent measurements from interacting areas, under certain experimental conditions, and it is found that the partial correlation of *X*_1_ and *X*_2_ given *X*_3_ is not statistically significantly different than zero, which could lead to the suggestion of Fig. 2*C*. Even if it is assumed that *1*) the measurements adequately represent relevant population activity and *2*) no other brain areas affect this relationship, it is extremely difficult to distinguish a small partial correlation from a zero partial correlation, and the usual interpretation of Fig. 2*C* corresponds to zero partial correlation. For this reason, the interpretation of graphical models requires care: when authors use a graphical model to draw conclusions, they should keep in mind their merger of small and zero partial correlations. Helpful discussions of useful methods for determining graphical models, e.g., Glymour et al. (163) sometimes appear to ignore this point or minimize its importance.

Causal manipulation can be provided by optogenetic stimulation, which has been a spectacular advance, yet requires care to avoid misleading results (164, 165). One common concern is an imperfect match between the targeted population activity and the activity of cells actually affected. In causal inference, well-defined manipulations that only approximate an ideal manipulation are known as instrumental variables (160, 161). In some cases, estimated effects can be adjusted to estimate the effects that would have occurred under the ideal manipulation (here, the ideal stimulation of the targeted population). The use of optogenetic manipulations as instrumental variables has been discussed by Segala and Looger (166) and Jiang et al. (167). Causal inference for neuroscience has been discussed in general terms by Biswas and Shlizerman (168) and by Marinescu et al. (169).

Causal effects are often depicted using directed graphs. An important caveat, however, is that in representing causal effects based on causal analysis, the directed graphs are restricted to being directed *acyclic* graphs (DAGs), which means they do not contain paths leading from a node through other nodes and back to the starting node. This restriction of causal graphs to DAGs rules out recurrent relationships.

## DISCUSSION

Our guide for data analysts wishing to identify interactions among neural populations has stressed analytical frameworks and statistical issues because progress requires both deployment of effective data analysis tools and appreciation of their inferential roles. There is a tension here, however, between powerful methods on the one hand, and a series of statistical caveats, on the other, caveats that can sometimes seem dishearteningly conservative especially when contrasted with the more ambitious culture of computer science that dominates machine learning (170).

The prevailing agnosticism of statistics toward theoretical implications of data-driven results is most apparent in statistical causal analysis where, as described in *Causality*, causal statements are said to concern “effects of causes” as opposed to “causes of effects.” Although the most important issue in causal reasoning, the potential influence of confounding variables, arises repeatedly in many contexts, it is, at least conceptually, widely appreciated by experimental investigators and is often addressed as a key discussion point in scientific reports. In addition, the flip side of the effects-of-causes versus causes-of-effects dichotomy is that the effects of causal manipulations resulting from the “gold standard” of randomized controlled trials, while crucial, are rarely fully satisfactory for scientific pursuit. This is clearest in clinical studies. For example, randomized controlled trials demonstrated the effectiveness of lithium in treating bipolar disorder long before the emergence of substantial knowledge about potential mechanisms and, while evidence of effectiveness has strengthened (171), even now mechanisms are not well understood (172). It is thus useful to distinguish causal evidence (delivered through causal analysis, as in randomized trials) from causal explanation, a term that may better capture the goals of neuroscientific investigations (e.g, Ref. 173; for philosophical discussion see Refs. 174 and 175). Furthermore, because causal analysis based on directed acyclic graphs rules out recurrence, causal explanations for recurrent circuits will have to go beyond standard frameworks for identifying causal evidence. Theoretical models, which have proven value in providing plausible mechanistic explanations that can inform understanding and guide future experimentation (176), will surely continue to play a role, and we may get closer to satisfying accounts of circuit behavior as suggested causes of effects are layered onto demonstrated effects of causes.

More generally, scientific articles often devote considerable space to post hoc explanatory hypotheses, stories that aim to synthesize and interpret results in terms of presumptive causal mechanisms. Such narratives are essential, even treasured components of the process. Still, it is important to recognize them as speculating about causes of effects, which is different from stating accurate interpretations of observed results. Scientific conclusions must combine preexisting knowledge and assumptions with data analyses based increasingly on advanced methods that involve subtleties of reasoning. This was a major motivation for our emphasis on statistical issues. As emerging technologies create more refined and detailed data, the concepts invoked here will likely endure by suggesting principled paths toward new methodologies.

## GRANTS

This work was supported by National Institute of Mental Health and Neurosciences Grant RO1 064537.

## DISCLOSURES

No conflicts of interest, financial or otherwise, are declared by the authors.

## AUTHOR CONTRIBUTIONS

R.E.K. conceived and designed research; H.B., M.O., Q.X., and K.U. analyzed data; H.B., Q.X., and K.U. prepared figures; R.E.K. drafted manuscript; R.E.K. edited and revised manuscript; R.E.K., H.B., M.O., Q.X., and K.U. approved final version of manuscript.
